# Reply

**Published:** 2010

**Authors:** Bashir A. Laway, Abdul H. Zargar

**Affiliations:** Sher-i-Kashmir Institute of Medical Sciences, Srinagar, Jammu and Kashmir, India

In response to the comments by Dr. Aggarwal, our patient had a last childbirth two years before and was not symptomatic during these two years, so the chance of it being due to peripartum cardiomyopathy does not arise. We do not agree with the author that the patient could have subclinical hypothyroidism precipitated by peripartum events. The patient did not have subclinical hypothyroidism (where we expect a normal T4 and mild elevation of TSH); in fact, she had severe secondary hypothyroidism as a part of panhypopituitarism because of Sheehan syndrome. Regarding details of antitubercular treatment, the patient was put on category 1 treatment consisting of isoniazid, rifampicin, pyrazinamide and ethambutol in recommended dosages. Yes, we believe that secondary adrenal insufficiency was precipitated by rifampicin, but we do not think cardiomyopathy was precipitated by antitubercular treatment, considering the known side effect profile of these drugs.

